# Serum amyloid A and other clinicopathological variables in cats with intermediate- and large-cell lymphoma

**DOI:** 10.1177/1098612X221135118

**Published:** 2022-11-23

**Authors:** Luca Schiavo, Petros Odatzoglou, Cassia Hare, Tim L Williams, Jane M Dobson

**Affiliations:** Department of Veterinary Medicine, University of Cambridge, Cambridge, UK

**Keywords:** Serum amyloid A, SAA, lymphoma, prognosis

## Abstract

**Objectives:**

Serum amyloid A (SAA) concentrations are increased in cats with lymphoma vs healthy cats; however, the association between SAA concentrations and prognosis in cats with lymphoma is unclear. The aim of this study was to evaluate if SAA concentrations were different in cats with nasal vs non-nasal lymphoma, if SAA concentrations are prognostic in patients treated with high-dose chemotherapy and if SAA concentrations are correlated with other clinicopathological variables.

**Methods:**

Cats diagnosed with intermediate- or large-cell lymphoma between 2012 and 2022 with SAA concentration data available were included. Associations between tumour site (nasal vs non-nasal), stage, response to treatment and SAA concentration were evaluated using non-parametric statistics. Associations between SAA concentrations and stage with survival time were evaluated using Cox regression analysis. Patients with nasal tumours and those not receiving high-dose chemotherapy were excluded from the survival analyses.

**Results:**

Thirty-nine cats were included. Median SAA concentrations were significantly higher in non-nasal compared with nasal lymphoma (42 µg/ml [range <0.3–797] vs <0.3 µg/ml [range <0.3–0.9]; P = 0.026). SAA concentrations did not correlate with tumour stage. Median survival time for patients with non-nasal tumour and undergoing chemotherapy was 49 days (range 2–1726). Responders had a better median survival time than non-responders (273 days [range 43–1728] vs 39 days [range 2–169]; P <0.001), whereas SAA concentrations were not associated with survival time. Lower haematocrit at presentation was associated with a reduced median survival time (P = 0.007).

**Conclusions and relevance:**

In the population examined, no correlation between serum concentration of SAA and prognosis in patients with lymphoma was identified, while low haematocrit and lack of response to treatment were both found to be associated with survival time. SAA concentrations were elevated in patients with non-nasal lymphoma vs patients with tumours confined to the nasal cavity.

## Introduction

Lymphoma is the most common haematopoietic neoplasm in cats, accounting for approximately 30% of all feline malignancies.^1,2^ Histologically, feline lymphoma can be classified as low, intermediate (IGL) or high grade (HGL), with IGL and HGL accounting for 35% and 50%, respectively, of all lymphomas in this species.^3^ Another, more aggressive subtype of HGL, called large granular lymphocyte lymphoma, linked to cytotoxic T or natural killer (NK) lymphocytes, has also previously been documented.^4,5^

Various protocols for the treatment of feline lymphoma have been described, with COP (cyclophosphamide, vincristine and prednisolone) or CHOP (cyclophosphamide, doxorubicin, vincristine and prednisolone) being the most widely used.^1,6–8^ Alternatively, more recent literature has suggested that a lomustine-based protocol is a viable alternative as a first-line and rescue treatment for cats with this malignancy.^9–11^ Median survival times in cats with lymphoma range from 27 to 955 days, with a response rate to chemotherapy ranging from 22% to 95%.^1,6,7,12^ Compared with canine lymphoma, in which several prognostic factors such as the immunophenotype and the stage of the disease have been identified, in cats the response to treatment remains the most significant factor associated with survival time.^1,6,7,13–15^ Other previously reported negative prognostic factors in cats include central nervous system (CNS) or bone marrow involvement, clinical substage b, granular cell morphology and anaemia.^1,4,7,16^ In contrast, disease confined to the nasal cavity is considered to have a better prognosis.^6,12,17^

Serum amyloid A (SAA) is a major acute phase protein (APP) in humans and cats.^18,19^ The protein is mainly produced by the liver under the influence of inflammatory cytokines such as interleukin (IL)-1 and IL-6, and tumour necrosis factor-alpha in response to inflammation or tissue damage.^20,21^ The role of SAA in the inflammatory response is not completely understood; however, previous literature suggests that the protein acts as an inflammatory mediator, particularly increasing cytokine production by monocytes and macrophages.^21–23^ SAA concentrations were found to be increased in several inflammatory and neoplastic diseases in humans and cats, and were recognised as a marker of distant metastatic disease in people with different neoplastic diseases.^24–26^ Additionally, in cats, SAA concentrations are an independent prognostic marker in patients with different diseases, including different types of neoplasia.^27^ SAA concentration is higher in cats with lymphoma vs a healthy population and decreased in patients achieving remission after undergoing chemotherapy.^28^ However, to our knowledge, no previous studies have studied the prognostic value of SAA concentrations in cats with lymphoma before undergoing chemotherapy.

The primary aim of the study was to correlate the SAA concentration with lymphoma in different tumour locations (nasal vs non-nasal), with tumour stage and with survival time. A secondary aim was to correlate SAA concentrations with other clinical and pathological variables, which were previously advocated as potential prognostic factors in other studies, in the same cohort of patients.

## Materials and methods

The medical records of a university referral hospital were reviewed between January 2012 and March 2022 for cats diagnosed with lymphoma. Patients were included if they had a cytological or histological (± immunohistochemistry) diagnosis of intermediate-to-large-cell lymphoma. Patients with a diagnosis of small-cell or large granular-cell lymphoma were excluded from the study. SAA concentration data were retrieved from the previous medical history or measured using archived frozen (–80^°^C) samples obtained at the time of presentation. SAA concentrations were measured on an Olympus AU400 or AU480 analyser using a human immunoturbidimetric assay previously validated for use in cats.^29^ The laboratory reference interval (determined as part of an internal validation study) for SAA concentration was <0.5 µg/ml and the limit blank of the assay was 0.3 µg/ml.

Neutrophil count, haematocrit (Hct) and serum albumin at presentation were also recorded. Patients were excluded if they received any treatment with chemotherapy, steroids, radiation therapy or surgery before SAA measurement. Follow-up information was retrieved from the medical records of the hospital or by contacting the referring veterinary surgeons. Only patients that underwent full staging, including thoracic (thoracic radiographs or thoracic CT), abdominal (abdominal ultrasound [US]) imaging and fine-needle aspiration of the spleen and liver were included when evaluating the association between tumour stage and SAA concentrations. Based on these findings, patients were retrospectively staged according to a previously described staging system (Table 1).^30^ The number of sites affected was also recorded. The time from treatment initiation to restaging was based on clinician preference. Patients were divided into two groups based on clinical response: responders (partial or complete remission) and non-responders (stable disease, progressive disease). Response to treatment was classified as complete remission (CR) if a complete disappearance of the visible disease was noted; partial remission (PR) if the lesion was reduced by at least 30% of its initial size but had not completely disappeared; stable disease (SD) if the disease had either reduced by less than 30% or increased up to 20%; or progressive disease (PD) if the disease had increased by more than 20%. Chemotherapy adverse events and their grade were classified according to the Veterinary Cooperative Oncology Group criteria.^31^ Median survival time was measured from the time of diagnosis to the time of death for any cause. Patients that did not receive high-dose chemotherapy, patients diagnosed in 2022 (due to short follow-up time) and patients with nasal lymphoma were excluded from the response-to-treatment and survival analysis. Exclusion criteria for the single groups analysed are summarised in Table 2.

**Table 1:** Clinical staging system for patients with lymphoma (based on Mooney and Hayes)^30^

Staging system for feline lymphoma
Stage 1
• A single tumour (extranodal) or single anatomical area (nodal), including primary intrathoracic tumours
Stage 2
• A single tumour (extranodal) with regional lymph node involvement • Two or more nodal areas on the same side of the diaphragm • Two single (extranodal) tumours with or without regional lymph node involvement on the same side of the diaphragm • A resectable primary gastrointestinal tract tumour, usually in the ileocecal area, with or without involvement of associated mesenteric nodes only
Stage 3
• Two single tumours (extranodal) on opposite sides of the diaphragm • Two or more nodal areas above and below the diaphragm
Stage 4
• Stages 1–3 with liver and/or spleen involvement
Stage 5
• Stages 1–4 with initial involvement of the central nervous system or bone marrow, or both

**Table 2:** Exclusion criteria for single groups of analysis

Analysis	Exclusion criteria	No. of patients (n = 39)
SAA in patients with nasal vs no nasal lymphoma	Whole population was included	39
SAA compared with patients’ Hct, neutrophils and serum albumin at presentation	Whole population was included	39
SAA compared with stage	Patients without thoracic and abdominal imaging were excluded	16
SAA compared with median survival time	Patients with nasal lymphoma and patients that received palliative treatment only were excluded; patients diagnosed in 2022 were excluded	25
Serum Hct, neutrophil count and serum albumin compared with median survival time	Patients with nasal lymphoma and patients that received palliative treatment only were excluded; patients diagnosed in 2022 were excluded	25

Patients with small-cell or large granular-cell lymphoma and patients that received steroids before the diagnosis were excluded from the initial population examined

SAA = serum amyloid A; Hct = haematocrit

Statistical analysis was performed using commercially available statistical software (SPSS version 25.0; IBM). For statistical purposes, a SAA concentration below the limit of blank of the assay (0.3 g/l) was assigned an arbitrary value of 0.15 mg/l. The Kruskal–Wallis or Mann–Whitney U-test was used to compare SAA concentrations between groups (stage and site). Spearman’s correlation coefficient was used to evaluate associations between continuous variables. Kaplan–Meier survival curves were constructed to calculate the median survival times of different groups, with survival between different groups compared using the log-rank test. Univariable Cox regression analysis was used to evaluate the association between SAA concentration, stage, site (nasal vs non-nasal) and response to treatment (responder vs non-responder) with overall survival time (all-cause mortality). Data are presented as median (range), unless otherwise specified, and a *P* value <0.05 was considered to be statistically significant.

## Results

### Patient characteristics

During the study period, a total of 39 cats met the inclusion criteria. Domestic shorthair was the most represented breed (n = 31), followed by domestic longhair (n = 1), Tonkinese (n = 1), Norwegian Forest Cat (n = 1), British Shorthair (n = 1), Ragdoll (n = 1), Russian Blue (n = 1), Siamese (n = 1) and Birman (n = 1). The median age of the population was 9 years (range 3–17). Male neutered cats were the most common (n = 28), followed by female neutered (n = 10) and female entire (n = 1). Feline immunodeficiency virus and feline leukaemia virus status was available in 24 cases, and was negative in all tested cases.

Alimentary lymphoma was the most common anatomical classification (n = 20), followed by renal (n = 7), nasal (n = 5), mediastinal (n = 2), splenic (n = 2), CNS (n = 1), skin (n = 1) and submandibular lymph node (n = 1). Lymphoma was diagnosed in more than one site in 28 cases, of which locoregional lymph nodes were the most common site involved (n = 21), followed by the liver (n = 7), spleen (n = 5), kidneys (n = 4), bone marrow (n = 2) and lungs (n = 1), and suspected myocardium (n = 1).

### Diagnosis

Diagnosis was achieved by cytology alone in 21 cases and by histology alone in six. Diagnosis was confirmed by cytology and histology in 12 cats. The lymphoma was classified as intermediate cell in seven cases and as large cell in the remaining 32. The immunophenotype was available in 11 cases, and was assessed by immunohistochemistry in nine cases and by flow cytometry in two. Immunophenotype was characterised as a B-cell neoplasia in five cases, T-cell neoplasia in four cases and as showing an aberrant, suspected NK immunophenotype in two. In the first case, the diagnosis of an NK immunophenotype was suspected as, on flow cytometry, the majority of gated cells lacked expression of any of the tested markers (CD3, CD4, CD5, CD8, CD14, CD21) other than CD18. In the second case, on immunohistochemistry, the neoplastic cells were negative for CD3 and CD79a (these were the only markers tested).

Thoracic imaging was performed in 26 cases; by CT scan in five cases and by thoracic radiographs in the remaining 21. Abdominal US was performed on 32 patients. Sixteen of these patients underwent US-guided fine-needle aspiration of the spleen and liver. Patients were classified as stage 1 in two cases, stage 2 in three, stage 3 in two, stage 4 in six and stage 5 in three.

### Treatment and survival

Of the entire population examined, 32 patients received chemotherapy alone, three patients radiation therapy alone, three were treated with palliative prednisolone only, and one received radiation therapy and chemotherapy. Of the patients treated with chemotherapy alone, 26 underwent a high-dose COP protocol and six received lomustine and prednisolone. One patient (with nasal lymphoma) that received radiation therapy and chemotherapy was treated with lomustine. The median number of chemotherapy doses administered in patients undergoing a COP protocol was five (range 1–12), while it was two (range 1–5) in the patients receiving lomustine. Lomustine (Bova Specials UK) was given at 10 mg/cat every 4–5 weeks in all cases. Data regarding the treatment received are summarised in Table 3. Chemotherapy-correlated adverse events were reported in 15 patients. Of these, nine developed neutropenia, which was graded as grade 1 in four patients, grade 2 in three, grade 3 in two and grade 4 in one. Gastrointestinal side effects were reported in seven cats and included three cats with vomiting, graded 1 and 2, three cats with diarrhoea, which was graded as 2 in two cats and 1 in the other, one case of constipation and two cats with grade 3 anorexia. Radiation therapy was administered to four patients in total. All these patients were diagnosed with nasal tumours. In all patients, a hypofractionated (three fractions), manually planned protocol was performed. The median dose administered was 24 Gy (range 11.5–24). Response to treatment information was available for 29 patients undergoing chemotherapy alone. In these, CR was achieved in 11 cases, PR in four and PD in 14.

**Table 3:** Summary of the treatment received when the entire population was analysed

Treatment	No. of patients	Median dose administered	Median (range) no. of doses
Chemotherapy	32	–	–
COP	26	–	5 (1–12)
Lomustine + prednisolone	6	10 mg/cat	2 (1–5)
Radiotherapy	3	8 Gy	3
Radiotherapy + lomustine	1	8 Gy 10 mg/cat	3
Prednisolone alone	3	–	–

The overall response rate for the population receiving chemotherapy alone (PR and CR; all patients receiving chemotherapy) was 52%. The date of disease progression was recorded in 23/34 cats. The median time to first progression was 36 days (range 2–887). Rescue protocols were used in 14 cases. Lomustine was the most common rescue agent (10 cases), followed by COP (one case), L-asparaginase (one case), and vincristine and cytarabine (one case). Radiotherapy was used in one case. The median time to second progression was 20 days (range 2–481). Only one patient received a second rescue protocol with cytarabine and prednisolone and was euthanased 24 days later owing to progressive disease. At the time of writing, 33 patients were deceased. Of these, 31 died as a result of tumour-related causes, one died after trauma on day 750 and one died from heart failure due to a previously diagnosed restrictive cardiomyopathy at day 32.

When patients with nasal lymphoma and patients that did not receive chemotherapy were excluded (n = 25), the median survival time was 49 days (range 2–1726). Median survival time for cats with nasal lymphoma was 250 days (range 31–2422).

### Association between SAA and nasal vs non-nasal lymphoma, stage, number of sites and haematobiochemical variables

SAA concentrations were measured at the time of diagnosis in 22/39 cases and on a frozen stored sample in the remaining 17 cases.

Median SAA concentration for the entire population was 5.3 µg/ml and ranged from <0.3 to 796.7 µg/ml. SAA was elevated (>0.5 µg/ml) in 25 patients (64%). SAA concentrations were significantly higher in patients with non-nasal compared with nasal lymphoma (42 µg/ml [range <0.3–797; n = 33] vs <0.3 µg/ml [range <0.3–0.9; n = 6]; *P* = 0.026).

Median Hct was 26% (range 14–47%), and the median neutrophil count was 10 × 10^9^/l (range 2–27). Median serum albumin level was 25 g/l (range 17–38). When compared with other clinicopathological values, SAA concentration showed a weak positive correlation with neutrophil count (*r_s_* = 0.443; *P* = 0.005) and a weak negative correlation with serum albumin concentration (*r_s_* = −0.317; *P* = 0.049). There was no statistically significant correlation between SAA concentrations and Hct (*r_s_* = −0.109; *P* = 0.511).

**Figure 1:**
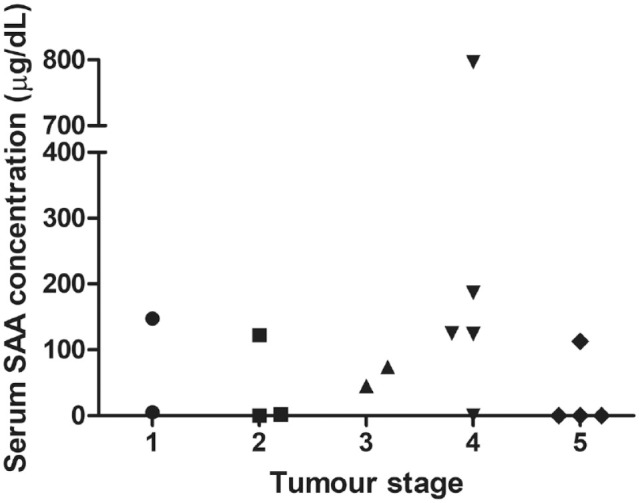
Serum amyloid A (SAA) values in different stages of the disease at presentation. No numerical difference was found between the different stages of the disease; however, no statistical analysis was performed owing to the low number in each group

After excluding patients that did not undergo full staging, 16 were available for analysis. SAA concentration of cats with different stages of lymphoma are shown in Figure 1. Owing to the low number of cases per stage group, statistical comparison was not possible; however, no obvious association between SAA concentrations and tumour stage was observed.

### Clinicopathological factors associated with survival

After the exclusion of patients with nasal lymphoma, patients that did not receive any treatment, and patients diagnosed in 2022, 25 patients were included in the survival analysis. Of these, 22 were treated with a COP protocol and three with a lomustine protocol.

In the univariable analysis, response to initial treatment was the only factor that was statistically significantly associated with survival time, with responders living significantly longer than non-responders (273 days [range 43–1728] vs 39 days [range 2–169]; *P* <0.001). SAA concentrations were not significantly associated with survival time (hazard ratio [HR] 0.996, 95% confidence interval [CI] 0.991–1.002; *P* = 0.18). Hct also tended towards a statistically significant association with survival time on univariable analysis (HR 0.952, 95% CI 0.898–1.009; *P* = 0.1).

In the multivariable analysis, increasing Hct (HR 0.034, 95% CI 0.827–0.970; *P* = 0.007) and response to treatment (HR 0.34, 95% CI 0.007–0.177; *P* <0.001) were found to be independently associated with increased survival time.

When the three cats treated with lomustine were excluded and only cats treated with COP were included in the analysis, SAA concentration was not statistically significantly associated with survival time (HR 0.998, 95% CI 0.991–1.005; *P* = 0.576), while increasing Hct (HR 0.896, 95% CI 0.827–0.970; *P* = 0.007) and response to treatment (HR 0.34, 95% CI 0.007–177; *P* <0.001) remained statistically significantly associated with increased survival time.

## Discussion

In this study, we evaluated the utility of SAA concentrations and other clinical and pathological variables as prognostic markers in a population of cats with intermediate-to-large-cell lymphoma. Feline nasal lymphoma tends to show a more localised behaviour than other forms of lymphoma in small animals. In most patients, the disease is confined within the nasal cavity, with only a minority showing systemic involvement.^12,32^ In our population, all cats apart from one showed a disease localised to the nasal cavity. Owing to this peculiar clinical behaviour, some authors have advocated the use of more localised radiotherapy treatment as an alternative to chemotherapy for the management of this neoplasia. So far, no studies have found any substantial difference in response between the use of local radiation therapy and systemic chemotherapy; however, in approximately 10–17% of patients treated with local therapy, distant progression of the disease was noted.^6,12,17,32^ For this reason, in our opinion, it is extremely important to have full knowledge of the systemic extent of the disease before choosing between the two treatments. In a study by Winkel et al,^28^ SAA concentrations were increased in cats with lymphoma, although no difference was found between different anatomical locations.^28^ In our population, the SAA concentration was increased in patients with non-nasal lymphoma vs those with nasal involvement. In our opinion, a low SAA value could reflect a less biologically aggressive disease, such as nasal forms of the tumour.

Previous studies in human medicine have proposed SAA concentrations as a marker of the extent of the disease in localised forms of haematological and solid neoplasia.^33,34^ Additionally, in the same study, an increase in SAA concentrations was found to be significantly correlated with a reduction in the median time of tumour progression.^33,34^

In our population, no correlation between the serum concentration of SAA and disease stage was observed. Larger studies regarding the value of SAA concentrations as a marker of tumour stage in cats are lacking. In a previous study in dogs, in which SAA was analysed in different neoplastic diseases, the serum concentration of the protein was not different between patients with lymphoma and leukaemia.^35^ It is therefore plausible that SAA may be correlated with specific locations of the disease associated with different biological behaviour, such as nasal forms of the tumour, but does not give any additional information regarding disease stage. However, the number of patients in our staging group was small, with a low number of patients divided into the different groups; therefore, larger studies are necessary to confirm this finding. Additionally, it would be interesting to see if an increase in SAA would differentiate between a localised form of nasal lymphoma and systemic forms of the disease. However, our population of cats with nasal lymphoma was too small to perform this analysis.

Furthermore, in our population, SAA concentration was not associated with prognosis. To our knowledge, no previous studies have investigated the role of SAA as a prognostic marker in feline haematological malignancies. However, a previous study by Correa et al investigated the association of serum alpha 1-glycoprotein concentrations – another APP – with prognosis in cats with lymphoma, although no correlation was found.^36^ This is in contrast with previous literature on human solid neoplasia, where the serum concentration of SAA at presentation was correlated with a reduced time to progression and survival time.^27,37^

In the population examined, a low Hct and lack of response to treatment were the only factors statistically significantly associated with survival. Anaemia has previously been associated with a reduced time to progression and survival time in cats with lymphoma.^6,38^ In patients with lymphoma, anaemia could be secondary to bone marrow infiltration, local disease or chronic gastrointestinal bleeding, or it could represent anaemia due to the inflammatory disease. Instead, the response to treatment has previously been reported as the most reliable prognostic factor in patients with lymphoma in multiple studies.^4,7,12,39^

This study had several limitations, mainly due to its small size and retrospective nature. First, patient staging was not standardised; to include patients with complete staging, there was a marked reduction in the number of cases that could be included. Second, the number of rechecks and reassessments was not standardised in the examined population, which may have caused an alteration in the calculated time to progression and in the timing to assess the response to the treatment in this study. Additionally, owing to its retrospective nature, there may have been inconsistency in the reporting of the adverse events that occurred in the population. The final limitation is that SAA concentrations were measured at the time of diagnosis in only some patients and on the frozen sample in around 50% of cases.

To our knowledge, there are no published studies regarding the stability of SAA in frozen samples in cats. A recent study in dogs did not show a significant difference in SAA concentration in frozen samples after 3 and 6 months.^39^ A small pilot study undertaken in our laboratory has not identified any significant reduction in SAA concentrations in samples stored for between 1 and 5 years (n = 15, data not shown). Therefore, we do not believe that the use of frozen samples would have significantly confounded our data.

## Conclusions

SAA concentration does not appear to be of value as a prognostic marker in cats undergoing chemotherapy for non-nasal, intermediate-to-large-cell lymphoma. However, the SAA concentration was elevated in patients with non-nasal lymphoma vs patients with a nasal form of the tumour. Further prospective studies with a larger population are required to confirm these findings.
